# Primary healthcare system readiness for the prevention and management of non-communicable diseases in Nepal: a mixed-methods study

**DOI:** 10.21203/rs.3.rs-6755631/v1

**Published:** 2025-06-12

**Authors:** Chandani Singh Nakarmi, Sanju Bhattarai, Elizabeth C Rhodes, Meghnath Dhimal, Phanindra Prasad Baral, Bikram Poudel, Binuka Kulung Rai, Anupama Bishwokarma, Sushmita Mali, Asmita Adhikari, Aarati Dhakal, Alina Bharati, Sangita Manandhar, Surakshya KC, Sashi Silwal, Bikram Adhikari, Soniya Shrestha, Felix Teufel, Dinesh Timilsina, Yunika Acharya, Donna Spiegelman, Archana Shrestha

**Affiliations:** Dhulikhel Hospital-Kathmandu University Hospital; Dhulikhel Hospital-Kathmandu University Hospital; Emory University; Nepal Health Research Council; Ministry of Health and Population; Dhulikhel Hospital-Kathmandu University Hospital; Dhulikhel Hospital-Kathmandu University Hospital; Dhulikhel Hospital-Kathmandu University Hospital; Dhulikhel Hospital-Kathmandu University Hospital; Dhulikhel Hospital-Kathmandu University Hospital; Dhulikhel Hospital-Kathmandu University Hospital; Dhulikhel Hospital-Kathmandu University Hospital; Dhulikhel Hospital-Kathmandu University Hospital; Dhulikhel Hospital-Kathmandu University Hospital; Nepal Health Research Council; Dhulikhel Hospital-Kathmandu University Hospital; Dhulikhel Hospital-Kathmandu University Hospital; Emory University; Dhulikhel Hospital-Kathmandu University Hospital; Dhulikhel Hospital-Kathmandu University Hospital; Yale School of Public Health; Dhulikhel Hospital-Kathmandu University Hospital

**Keywords:** Non-communicable diseases, Primary healthcare system, Service readiness, PEN program

## Abstract

**Background:**

Non-communicable diseases (NCDs) contribute to two-thirds of Nepal’s total deaths. In 2016, Nepal adopted the World Health Organization’s Package of Essential Non-Communicable Disease Interventions (WHO-PEN) to curb the growing burden of non-communicable diseases (NCDs). This study evaluated the primary healthcare system’s readiness for the prevention and management of non-communicable diseases (NCDs), including cardiovascular diseases (CVDs), diabetes mellitus (DM), and chronic respiratory diseases (CRDs), and investigated factors associated with NCD-specific service readiness.

**Methodology:**

We employed a convergent parallel mixed-methods study design. We adapted the WHO Service Availability and Readiness Assessment (SARA) tool and assessed 105 primary healthcare facilities, which were selected using a multistage stratified random sampling approach. We performed a weighted descriptive analysis and fitted survey-weighted multivariable linear regression to identify factors associated with NCD-specific service readiness. Simultaneously, we conducted 23 key informant interviews with health authorities and 47 in-depth interviews with health service providers involved in the PEN implementation. All interviews were audio recorded, transcribed verbatim, and analyzed using a thematic approach.

**Results:**

The overall NCD service readiness score for primary healthcare facilities was highest for CVDs at 48.4 (95% CI: 43.2–53.6), followed by DM at 40.8 (95% CI: 34.5–47.2), and CRDs at 34.8 (95% CI: 29.2–40.5). Primary Healthcare Centers (PHCCs) had higher NCD service readiness than health posts. In regression analysis, we found that primary healthcare facilities located in hilly regions and imposing user fees for some NCD services had significantly higher NCD-specific service readiness compared to those in the mountainous areas and those not imposing user fees, respectively. Qualitative findings revealed that higher NCD service readiness in PHCCs and certain regions was due to better infrastructure, training opportunities, accessibility to medicines and equipment, and social health insurance schemes. High staff turnover and limited supply of NCD drugs and equipment hindered NCD service delivery, particularly in health posts and remote regions.

**Conclusion:**

Primary healthcare facilities in Nepal lack equipment, medicines, trained staff, and guidelines for NCD management. The government of Nepal could enhance NCD-specific service readiness by equipping health service providers with medical supplies and building their capacity through regular PEN training and peer coaching sessions.

## Introduction

Non-communicable diseases (NCDs) account for 71% (41 million) of all global deaths, and three-fourths (77%) of these deaths occur in low- and middle-income countries (LMICs) [1]. Nearly 80% of the NCD morbidities in the world are from one of the four major NCDs: cardiovascular diseases (CVDs), chronic respiratory diseases (CRDs), diabetes mellitus (DM), and cancers [2].The probability of dying between the ages of 30 and 70 years from one of these NCDs is 17.8% globally and 21.5% in Nepal [3]. The overall prevalence of NCDs among Nepalese individuals aged 15–69 years is 55.4%. The burden of hypertension (27.3%) [4], type-2 DM (8.5%) [5], and chronic obstructive pulmonary disease (COPD) (11.7%) [6] is substantial. NCDs in Nepal contribute to 61.2% of disability-adjusted life years (DALYs) in Nepal [7] and pose a significant burden on the health system of Nepal, which is primarily financed by out-of-pocket payments [8,9]. More than 50% of the total out-of-pocket expenditure for health is spent on NCDs [10].

In response to the large and increasing burden of NCDs, the government of Nepal implemented the National Multisectoral Action Plan (2014–2020) [11] and later endorsed the WHO’s Package for Essential Non-Communicable Disease (hereafter referred to as the PEN program) in 2016 [12]. The PEN program consists of evidence-based, cost-effective interventions for the prevention and management of hypertension, diabetes, asthma, COPD, and breast and cervical cancer [12]. In 2017, the government of Nepal piloted the PEN program in two districts and gradually expanded it to 31 of the 77 districts in 2018 and 2019 [12,13]. A feasibility study in PEN-piloted districts highlighted several gaps in health system preparedness for PEN program initiation, namely insufficient training of health workers, lack of medical supplies, and limited diagnostics services, and underscored the need to strengthen the healthcare delivery system before scaling the program at the national level [13].

For LMICs like Nepal, the primary healthcare system is the preferred choice for delivering basic health services, including those for NCDs [14]. A few studies in Nepal have reported the overall NCD service readiness of health facilities at the primary, secondary, and tertiary levels; however, no study has comprehensively evaluated the readiness of the primary healthcare system for managing NCDs [9,15]. This knowledge gap can thwart the government’s effort towards achieving Sustainable Development Goal 3.4, i.e., reducing premature mortality from NCDs by one-third by 2030 [16]. Our study aims to evaluate the readiness of the primary healthcare system for the prevention and management of CVDs, DM, and CRDs, and identify factors associated with each disease-specific service readiness. The study findings will help the government and policymakers develop strategies to improve the readiness of primary healthcare facilities, which is crucial for the successful implementation and scaling up of the PEN program in Nepal, as well as in other low- and middle-income countries (LMICs) with similar health system contexts.

## Methodology

### Study design

**Figure 1:**
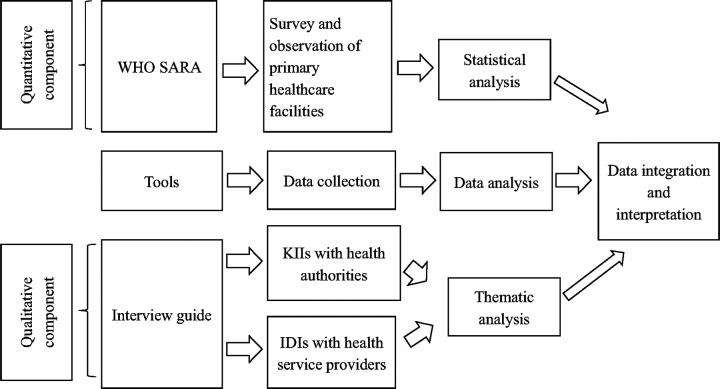
Convergent parallel mixed methods study design WHO SARA: World Health Organization Service Availability and Readiness Assessment, IDIs: In-Depth Interviews, KIIs: Key Informant Interviews

We conducted a convergent parallel mixed-method study to evaluate the readiness of the primary health system to implement the PEN program in Nepal. The qualitative and quantitative data were collected in parallel, analyzed separately, and then merged for interpretation [17]. We quantitatively assessed the readiness of primary healthcare facilities for the screening and management of three major NCDs: diabetes mellitus (DM), cardiovascular diseases (CVDs), and chronic respiratory diseases (CRDs) [18]. Additionally, we conducted qualitative interviews with health authorities and health service providers involved in the PEN program implementation (Figure 1).

### Study setting

Nepal is a lower-middle-income country in Southeast Asia with an area of 147,516 km^2^ [19] and a total population of 29,112,480 [20]. It is administratively divided into 7 provinces, within which lie 77 districts, each subdivided into rural and urban municipalities. All health-related activities are implemented under the close supervision of three tiers of government: federal, 7 provincial, and 753 local governments [9,21]. The federal and provincial governments oversee secondary and tertiary level healthcare, while local governments are responsible for providing primary health services. The primary-level healthcare is provided through a network of 189 Primary Health Care Centers (PHCCs) and 3,794 health posts [22]. The PEN program in Nepal focuses on improving NCD management through early detection in primary healthcare facilities, including health posts and PHCCs. District and provincial hospitals at the secondary level, as well as tertiary-level specialized hospitals, serve as referral points for managing NCD-related emergencies and complications [23].

### Quantitative component

#### Sampling

We calculated the minimum required sample size to estimate the NCD service readiness in primary healthcare facilities using the one-sample mean formula, $n=\frac{Z_{\alpha /2}^{2}\times \sigma^{2}}{d^{2}}$. Assuming a 95% confidence level, a standard deviation (σ) of 17.6 units based on the 2015 Nepal health facility survey mean diagnostic index for diabetes services for public facilities [24], and a margin of error of ± 3.5 units, the minimum required sample size is 97 [25]. After adding an 8% non-response, the calculated sample size was 105.

**Figure 2:**
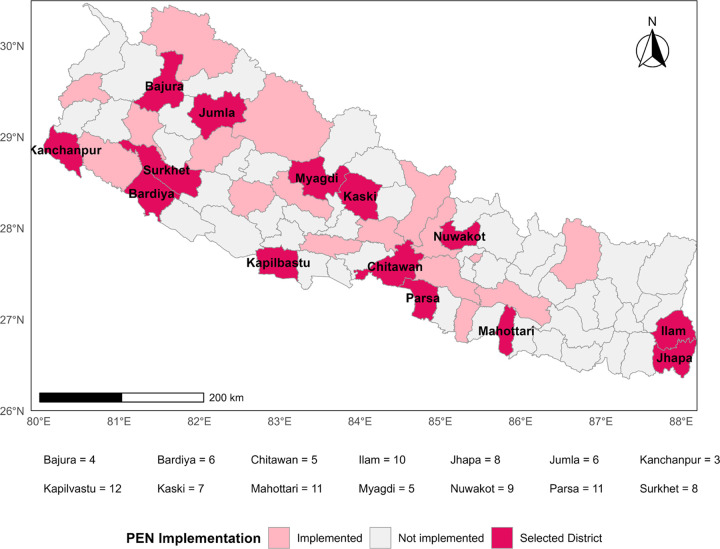
Sampling distribution of primary healthcare facilities across selected PEN-implemented districts of Nepal

A multistage stratified random sampling was applied to select primary healthcare facilities from the sampling frame of 31 districts implementing PEN as of 2018–2019, distributed across all 7 provinces. In the first stage, we randomly selected 14 PEN-implemented districts by selecting two districts from each province using the STATA software [26]. In the second stage, all primary healthcare facilities from the sampled districts (653 health posts and 41 Primary Health Care Centers, or PHCCs) were listed and used as the sampling frame. We stratified health facilities into rural and urban areas, randomly selecting PHCCs and health posts proportionate to their numbers within each stratum. PHCCs were oversampled as they represented only 5.9% of the total primary healthcare facilities in the sampled districts. The final sample selected for the survey consisted of 71 health posts and 34 PHCCs, totaling 105 primary healthcare facilities (Figure 2).

#### Data collection tools and techniques

We collected data between August 2020 and April 2022. We adapted the World Health Organization’s Service Availability and Readiness Assessment (WHO-SARA) tool, following the guidelines outlined by the national PEN manual of Nepal, to assess the readiness of primary healthcare facilities to deliver NCD-related services [18]. The WHO-SARA tool has been previously used in Nepal and several countries to assess the NCD-specific service readiness [9,27–30]. We used the WHO-SARA health facility inventory tool to collect information on the availability of NCD-specific equipment, drugs, and guidelines, and a health service provider questionnaire to obtain information related to PEN training, user fees, and monitoring activities by the higher government. To collect data, eight trained research assistants contacted primary healthcare facilities in charge via telephone calls to set up on-site visits at their convenience. The research assistants explained the purpose of the visit, obtained written informed consent, and conducted face-to-face interviews using a paper-based questionnaire. They also verified service providers’ responses by directly observing facilities for equipment, drugs, and guidelines.

### Measurement of variables

#### Outcome variables

We assessed three outcome variables in this study: the readiness of primary healthcare facilities for managing diabetes mellitus (DM), cardiovascular diseases (CVDs), and chronic respiratory diseases (CRDs). We calculated a mean composite NCD-specific service readiness score based on the availability of tracer indicators identified in the World Health Organization’s SARA reference manual. The selected SARA indicators were also verified with the national PEN manual and Nepal’s national essential drug list [31]. Based on the WHO-SARA manual, we grouped tracer items into three domains: i) staff training and guidelines, ii) equipment and diagnostics, and iii) essential medicines. We ascertained readiness in each of the domains described in Table 1. The value “1” was assigned if tracer items were present, and “0” was assigned if items were unavailable in primary healthcare facilities. The mean readiness score for each domain was calculated by summing the item values and then dividing the sum by the number of items, followed by multiplication by 100 (Supplementary Table 1). The overall NCD-specific service readiness ranged from 0 to 100. A higher readiness score indicated better service readiness for managing non-communicable diseases (NCDs).

#### Explanatory variables

We ascertained the association of NCD-specific service readiness with the type of primary healthcare facility (PHCCs/health posts), area (rural/urban), region (mountain/hills/lowlands), and the applicability of a separate user fee for NCD services (yes/no). The explanatory variables were selected based on the literature review [9,27,32]. Research assistants interviewed health service providers to obtain information about user fees and monitoring and supervision visits for the PEN program. Health service providers’ responses were also further verified with charge sheets and registers, if available.

#### Data management and analysis

We checked for missing entries on the same day that the data were collected. We resolved the problem with missing entries through a follow-up visit or phone call with the health facility in charge. Data were entered into online forms created in the Kobo Toolbox [33], exported into Excel, and imported to R version 4.3.2 [34] for cleaning and analysis.

During the analysis, the sample statistics were weighted to reflect a nationally representative sample of primary healthcare facilities. The sampling weight, which is the inverse probability of selection of sample units, relied on stratification. First, district weight was calculated as the inverse of the selection probabilities of PEN-implemented districts for each province. Second, the weight for primary healthcare facilities was calculated based on their stratification by primary healthcare facilities’ setting (rural/urban) and type (health post/PHCC). Finally, the overall sampling weight was calculated as the product of two intermediate weights: the district weight and the weight of primary healthcare facilities.

The categorical variables were summarized using frequencies and percentages after applying sampling weights. NCD-service-specific readiness scores for diabetes, CVDs, and CRDs were expressed as means and 95% confidence intervals. We visualized readiness scores by primary healthcare facility type for each disease using bar plots, with error bars representing the 95% confidence interval (CI). We performed survey-weighted multivariable linear regression using the “svyglm” function from the survey package in R to determine the factors associated with NCD service readiness. The “svyglm” package in R enabled us to account for the complex survey design by incorporating survey weights, ensuring robust variance estimation. Results were reported as regression coefficients (β) with 95% confidence intervals (CIs). Statistical significance was set at p-value<0.05.

### Qualitative methods

#### Sampling

We purposively sampled health authorities involved in planning, implementing, and monitoring the PEN program, as well as health service providers who delivered PEN services at the primary healthcare level. We conducted 23 key informant interviews (KIIs) with health authorities (Supplementary Table 2) and 47 in-depth interviews (IDIs) with health service providers (Supplementary Table 3) who had received training in the PEN program. The sample size estimation for IDIs and KIIs was determined by code and meaning saturation [35]. The research team regularly conducted debriefing sessions to discuss data collection and saturation. We continued data collection until no new themes emerged in the qualitative data analysis.

#### Data collection

We developed semi-structured interview guides for key informant interviews (KIIs) with health authorities and in-depth interviews (IDIs) with health service providers following the WHO-SARA manual. The interview guides covered four main aspects of the WHO-SARA manual: human resources, guidelines, equipment, and medicines for NCD management. The guides were initially developed in English and then translated into Nepali, pilot-tested among two health service providers and one health authority of non-participating health institutions, and refined. More than half of the interviews were conducted in person (n = 41); the remaining interviews were conducted virtually via telephone (n = 10) or online using Google Meet (n = 19) due to COVID-19-related travel restrictions and social distancing requirements. The interviews were conducted by a research coordinator (CSN), who holds a master’s degree in public health, and two research officers (SM and SS), who hold master’s degrees in sociology, as well as two research assistants (AD and AB), who hold bachelor’s degrees in public health. All interviews were conducted in a private space in primary healthcare facilities or health office settings and recorded using a digital audio recorder. The average duration of interviews was 54 minutes (ranging from 23 to 77 minutes) for health authorities and 41 minutes (ranging from 22 to 56 minutes) for health service providers. Following each day of data collection, the research team discussed data emerging from interviews and revised interview guides as needed through an iterative process.

#### Data management and analysis

Research assistants transcribed all IDIs and KIIs verbatim in Nepali, and transcripts were compared against their respective audio files for accuracy and de-identified. We employed an inductive-deductive approach to thematic analysis [36,37], initially identifying themes from the qualitative data and subsequently organizing them according to the WHO SARA domains [18]. The research team reviewed one-third of transcripts in each category to identify issues in the data and developed separate codebooks for health authorities and health service providers. The codebooks included a list of codes, their definitions, and examples of code application, and were developed through an iterative process with inputs from an experienced qualitative researcher (ER). The Dedoose software (version 7.0.23) [38] was used to manage and code textual data. Initially, two coders separately coded three or more interviews in each category and matched them to calculate the percentage agreement. Any disagreements in code application were resolved through subsequent discussions, followed by iterative revisions to the codebook. After obtaining a minimum of 80% agreement, the coders independently coded the remaining interviews. The coders searched, coded, and categorized excerpts within the dataset, identifying a repetitive pattern of themes and discussing all emerging codes and meaningful patterns throughout the coding process. The research team conducted consecutive discussions to identify important emerging themes and mapped them to the WHO SARA framework [18].

#### Data integration

We applied a contiguous narrative approach, presenting qualitative and quantitative findings in separate sections of the results. The first half of the result section reports findings from primary health facility assessments, and the second half encompasses key findings from qualitative research [39]. The research team simultaneously reviewed quantitative statistical analysis and emerging themes from qualitative analyses to evaluate the congruence, divergence, or complementarity of the findings. Both qualitative and quantitative data were given equal priority during the study.

## Results

### Background characteristics and operational aspects of primary healthcare facilities in Nepal

Table 2 presents unweighted and weighted distributions of primary healthcare facility characteristics; all descriptions are based on weighted estimates to ensure national representativeness. Of the total 105 primary healthcare facilities, 94.1% were health posts. In terms of geographical distribution, more than half of the primary healthcare facilities were in rural areas (57.1%) and from the lowlands (52.4%). Nearly one-third (35.6%) of primary healthcare facilities offered 24-hour emergency services, typically operating for a median of 7 hours (Q_1_:7, Q_3_:7). A few (6.8%) primary healthcare facilities implemented social health insurance, and 29.7% charged separate user fees for some NCD services. The median distance to the nearest referral center was approximately 16 kilometers (Q_1_:9, Q_3_:25). Most (84.8%) of the primary healthcare facilities reported no monitoring and supervision visits for the PEN program (Table 2)

### Availability of basic diagnostic equipment, medicines, trained staff, and guidelines for the management of NCDs

**Figure 3:**
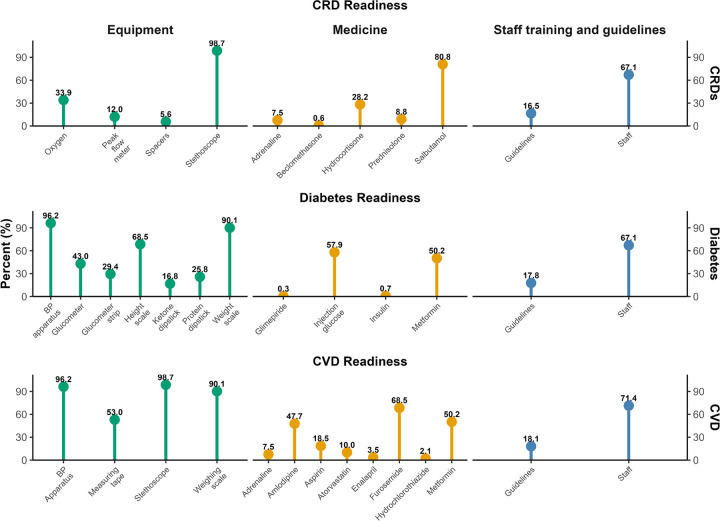
Weighted percentage availability of basic diagnostic equipment, medicines, guidelines, and at least one trained staff for the management of DM, CVDs, and CRDs in primary healthcare facilities (n=105) DM: Diabetes Mellitus, CVDs: Cardiovascular Diseases, CRDs: Chronic Respiratory Diseases

Most primary healthcare facilities had adult weighing scales (90.1%), blood pressure measuring apparatus (96.2%), and stethoscopes (98.7%). Glucometers, blood glucose test strips, and urine ketone dipsticks, which are specific for diagnosing diabetes, were available in 43%, 29.4%, and 16.8% of primary healthcare facilities, respectively. Peak flow meters and spacers for inhalers were available in only 12% and 5.6% of primary healthcare facilities for the diagnosis and management of CRD. Nearly half of the primary healthcare facilities (50.2%) used metformin and amlodipine (47.7%) for the management of diabetes mellitus (DM) and cardiovascular diseases (CVDs), respectively. Only 10% of primary healthcare facilities had atorvastatin for the prevention and management of cardiovascular diseases (CVDs). Most (80.8%) of the primary healthcare facilities had salbutamol tablets for the management of CRDs. The availability of trained staff (71.4%) and guidelines (18.1%) was highest for CVDs compared to CRDs and DM. Two-thirds (67.1%) of primary healthcare facilities had at least one trained health service provider for managing DM and CRDs (Figure 3).

### Readiness of the primary healthcare facilities for the management of NCDs

**Figure 4:**
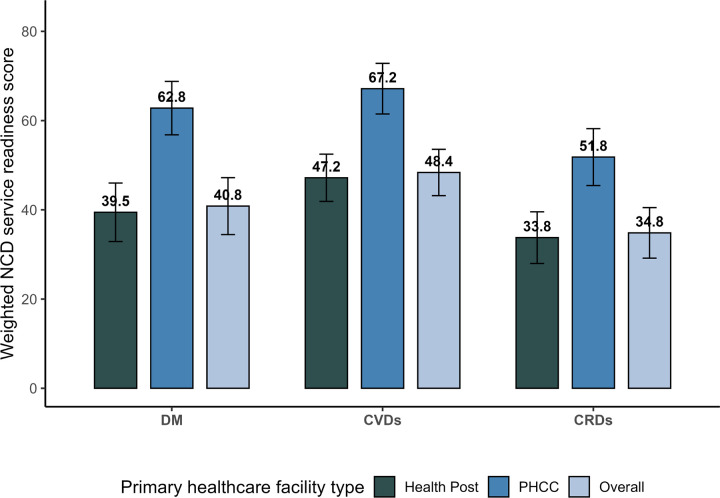
Readiness scores of primary healthcare facilities for the management of DM, CVDs, and CRDs (n=105) DM: Diabetes Mellitus, CVDs: Cardiovascular diseases, CRDs: Chronic Respiratory Diseases

Figure 4 illustrates NCD-specific service readiness across health posts, PHCCs, and overall primary healthcare facilities. The mean service readiness score for primary healthcare facilities was highest for CVDs at 48.4 (95% CI: 43.2–53.6), followed by DM at 40.8 (95% CI: 34.5–47.2), and CRDs at 34.8 (95% CI: 29.2–40.5). Overall, health posts had low service readiness for all NCDs: CVDs at 47.2 (95% CI: 41.9–52.5), DM at 39.5 (95% CI: 32.9– 46.0), and CRDs at 33.8 (95% CI: 28.0–39.6). Conversely, PHCCs, compared to health posts, demonstrated higher service readiness scores for CVDs (67.2, 95% CI: 61.5–72.8), DM (62.8, 95% CI: 56.8–68.8), and CRDs (51.8, 95% CI: 45.5–58.2).

### Factors associated with primary healthcare facilities’ readiness for the management of DM, CVD, and CRD

Table 3 shows that PHCCs had significantly higher service readiness scores compared to health posts for CVDs (β = 9.88, 95% CI: 0.37 to 19.34), DM (β = 11.90, 95% CI: 0.37 to 23.43), and CRDs (β = 13.18, 95% CI: 4.96 to 21.40). Compared to primary healthcare facilities in mountain regions, those in hills had significantly higher readiness for CVDs (β = 17.63, 95% CI: 10.18 to 25.08) and CRDs (β = 16.43, 95% CI: 9.36 to 23.50). Likewise, the primary healthcare facilities with the provision of separate fees for some components of NCD services had significantly higher readiness compared to those without user fees for CVDs (β=11.75, 95% CI:5.05 to 18.44), DM (β=14.21, 95% CI: 5.44 to 22.98), and CRDs (β=6.79, 95% CI: 0.74 to 12.84).

### Qualitative findings

We mapped the qualitative findings to WHO SARA domains and presented the key findings under three overarching themes: i) staff training and guidelines, ii) equipment and diagnostics, and iii) basic medicines. The qualitative findings explored the readiness of primary healthcare facilities for each SARA domain and explained key discrepancies in NCD-specific service readiness based on primary health facility type, region, and application of user fees. The quantitative and qualitative findings complement each other, reinforcing the overall results.

#### Staff training and guidelines

##### Availability of PEN-trained staff

None of the primary healthcare facilities had staff solely dedicated to the PEN program. Many primary healthcare facilities had one to three PEN-trained health service providers, but they managed multiple programs, such as maternal and child health, family planning, and communicable diseases. All participants unanimously regarded staff adjustment post-federalization as the principal reason for the shortage and inequitable distribution of PEN-trained staff in primary healthcare facilities and health offices.

“After federalization, there was an exodus of permanent staff to the northern region. Only half of the sanctioned positions in primary healthcare facilities are fufillled.”

-Public Health Inspector (HA2), Lowlands, District level

Most participants felt overburdened due to the staff shortage, and the addition of the PEN program further increased their responsibilities, compromising NCD service delivery. Some primary healthcare facilities and health offices hired untrained staff temporarily to offset the staff deficit but were unable to continue NCD services. Replacing vacant positions was particularly challenging for remote primary healthcare facilities, and even when new staff were hired, their retention was still uncertain.

“Temporary staff usually leave after a year of service, and we again have to recruit someone to fill in the vacant positions.”

-Public Health Section Officer (HA18), Hill, Provincial level

While many participants reported inadequate human resources in their offices, few primary healthcare facilities, mostly PHCCs, in the lowlands and hilly regions mentioned having adequate staff. One PHCC in the lowlands was in the process of being upgraded to the district hospital and was overstaffed. Regardless of having adequate and competent health service providers, many PHCCs were overburdened with high patient flow and could not offer detailed patient examination.

“Instead of 10 health workers, we have 25 health workers. We hired extra health workers on contract as our PHCC is upgrading to the district hospital.”

-Medical officer (HSP5), PHCC, Lowlands

In PHCCs, medical officers examined and prescribed medicines for NCD patients, and paramedics provided counseling. Some PEN-trained medical officers and paramedics also reported organizing coaching sessions for untrained staff. In a few instances, PEN orientations were also organized for community leaders and female community health volunteers. Some participants believed that medical officers in PHCCs were capable of NCD care provision even without PEN training. Health assistants and paramedics in PHCCs also felt competent and believed they could provide NCD services after completing the PEN orientation and peer coaching. Health service providers in health posts, on the other hand, were less receptive to PEN orientation and peer mentoring compared to service providers in PHCCs, and preferred waiting for PEN training to be provided.

“Not all health service providers in our health facility are trained, but they can handle NCD patients properly and provide NCD services as I have oriented them about PEN.”

-Medical officer (HSP5), PHCC, Lowlands

Overall, PEN training enhanced the capacity of health service providers to deliver NCD services, including diet education, blood pressure assessments, and the referral of emergency cases. Most of the PEN-trained health service providers were medical officers and paramedics. Nurses, not primarily involved in NCD patient examination and management, were less prioritized for PEN training. Even PEN-trained nurses were not providing NCD services as they had other duties and obligations.

“Although our nurses are PEN-trained, they are assigned responsibilities of birthing, antenatal care, and family planning; therefore, other paramedics look after NCD patients in outpatient departments.”

-Public Health Inspector (HSP19), Health post, Hill

##### Availability of NCD guidelines

Many health service providers found that having the PEN manual helped estimate CVD risks, perform clinical examinations, prescribe medicines, and refer patients. However, some health service providers didn’t receive hard copies of the PEN manuals, and a few who did misplaced or lost them when a natural disaster destroyed the facilities’ infrastructure.

“Previously, we had the PEN manual, but a landslide wrecked the health facility, and we lost the only guideline that we had.”

-Senior AHW (HSP13), Health post, Hill

Health service providers often cited the unavailability of a CVD risk estimation chart as the reason for not estimating the CVD risk in patients. A few proactive health workers printed digital copies of CVD risk estimation charts and used them further to calculate CVD risk among patients; however, these printed copies of CVD charts could be easily misplaced.

“Handouts can get misplaced. If we could have provided separate risk prediction charts like ip charts, it would have been much easier for health service providers.”

-Public Health Officer (HA4), District level, Lowlands

Some health service providers could refer to the PEN manual, especially when patient flow is low in the health facilities. Having a low patient flow provided them with time to refer to the guidelines when providing NCD services. A few health service providers mentioned keeping the PEN manual in the patient examination room for easy access, so they could refer to it whenever they had confusion and needed further help to make clinical decisions.

“I have a manual that was provided during the training session. I follow the manual for the diagnosis of patients.”

-Medical officer (HSP9), PHCC, Hill

#### Equipment and diagnostics

Many health service providers cited the availability of equipment, such as weighing scales, measuring tapes, blood pressure measurement sets, and stadiometers, in their primary healthcare facilities, while a few mentioned that they also lacked these basic instruments. Equipment, such as glucometers provided during initial training, was not maintained, and consumables, including glucose test strips and urine protein strips, were not supplied, or only expired strips were made available. Several health service providers mentioned having non-functional glucometers for blood sugar measurement. The unavailability of functional glucometers mostly disrupted the NCD services at health posts, as they were not equipped with laboratory analyzers.

“During my incumbency at this PHCC, neither glucometers nor blood glucose test strips were provided. From what I understand, they were once supplied, and there hasn’t been any maintenance, restocking, or follow-up since then; obviously, they didn’t last indefinitely.”

-Medical Officer (HSP9), PHCC, Hill

Few health service providers cited the increased availability of glucometers and blood glucose test strips from senior citizen programs for adults aged 70 years and older. Some primary healthcare facilities even charged minimal user fees to NCD patients for specific diagnostic and therapeutic procedures. This helped primary healthcare facilities to maintain the availability of NCD services, without running out of necessary equipment.

“We charge a nominal fee for blood glucose and lipid profile tests. This fee helps us maintain a consistent supply of reagents and kits to keep the lab operational.”

- Medical officer (HSP2), PHCC, Lowlands

In terms of medical supplies, PHCCs were comparatively more equipped than health posts. Few health service providers acknowledged the availability of sophisticated equipment, including electrocardiogram (EKG/ECG) machines, laboratory analyzers, and X-ray diagnostics.

“Equipment like an ECG machine, BP set, and analyzer is available to us; we can test blood sugar here in our primary healthcare facility.”

-Senior Auxiliary Health Worker, PHCC, Hill

The unavailability of a peak flow meter was one of the major complaints among the health service providers in PHCCs and health posts. Some health authorities mentioned that they had been provided with peak flow meters for the PEN trainees, who either misplaced or never used them due to their high operating costs. In the absence of peak flow meters, some healthcare service providers reported using stethoscopes and X-rays for diagnosing CRDs.

“A metered dose inhaler (MDI) and Drug Powdered Inhaler (DPI) are required to operate a peak flow meter, which costs nearly 450 to 550 Nepalese rupees, and it is challenging to manage these health care costs.”

-WHO PEN field coordinator (HA5), central level

The unavailability of diagnostic equipment in health posts and Primary Health Care Centers (PHCCs) led health service providers to resort to history-taking and physical examination for the diagnosis of NCDs.

“For NCD patients coming to our health facility, we usually monitor vital signs, obtain a history of symptoms onset, and prescribe medicines. We also need to ask questions on diet, frequency of lab investigations, and conduct key function tests among patients receiving medications, but it is not being brought into practice.”

-Health Assistant (HSP11), PHCC, Hill

#### Essential medicines

Many health authorities and service providers have reiterated the unavailability of essential medicines as a critical bottleneck for NCD service delivery. The primary healthcare facilities mostly provided freely available essential medicines like amlodipine, hydrochlorothiazide, atenolol, metformin, atorvastatin, and salbutamol tablets free of cost. However, the supply of these NCD drugs, particularly atorvastatin, was mostly irregular, discouraging NCD patients from continuing to seek services.

“When basic medicines like amlodipine and metformin are not available in the health post, patients visiting our health post get discouraged and directly visit higher centers for medicine refills.”

-Health Assistant (HSP25), Health post, Mountain

Many health service providers shared their dilemmas when following the PEN algorithms, as the national essential drug list does not cover all non-communicable disease (NCD) drugs mentioned in the PEN protocols. For instance, the PEN protocol recommends nebulization and administration of steroids and salbutamol via metered dose inhalers (MDIs) or drug-powdered inhalers (DPIs), but these drugs were not freely available. Health service providers in health posts were obliged to continue using phased-out medication or refer CRD patients to higher centers for further management. The situation was dire in remote and mountainous areas where NCD patients had limited accessibility to other healthcare institutions. Health service providers often felt helpless when NCD patients returned from their facilities without getting their medicines refilled.

“When doctors from higher referral centers prescribe metformin and glimepiride, we only provide metformin due to the unavailability of glimepiride.”

-Senior Auxiliary Health Worker (HSP7), Health post, Lowlands

Health service providers in PHCCs had better flexibility in writing prescriptions for NCD patients with social health insurance schemes; however, not all NCD patients visiting the PHCCs had health insurance. Some health service providers in PHCCs found it particularly challenging when some drugs for NCDs were available free of cost, while others were not. NCD patients not enrolled in health insurance were either prescribed freely available essential drugs or asked to purchase non-essential medicines from private drug outlets, depending on the patients’ affordability and accessibility. There were also some instances when PHCCs ran out of supplies and could not even offer NCD drugs through insurance and faced patients’ criticism. Some NCD patients even discontinued receiving health services from primary healthcare facilities due to the unavailability of NCD drugs.

“When basic medicines like amlodipine and metformin are not available in our health post, patients are discouraged from revisiting our health post… If they need those medicines in the future, they will directly go to higher centers, thinking that the medicines will never be available in our facility.”

-Health Assistant (HSP25), Health post, Mountain

Besides health insurance, the senior citizenship scheme also ensured access to NCD drugs for older adults (>70 years) visiting primary healthcare facilities. NCD drug procurement and supply chain management were also affected by federalism and COVID-19. Some participants reported an increased supply of NCD drugs by the local municipalities after federalism, while many pointed out that federalization only complicated the drug procurement process. Due to COVID-19, primary healthcare facilities reported a low stock of NCD drugs but increased supplies of thermometers, sanitizers, hand wash, and masks for COVID-19 prevention and control.

The province must purchase medicines in bulk, so the procurement process is complicated. In every tendering process, the province faces one or other legal issues, and the tendering process gets delayed by two to three months.”

-Public Health Inspector (HA2), District level, Lowlands

### Integrated results

The quantitative and qualitative findings together provided complementary insights into the readiness of Nepal’s primary healthcare system for the NCD prevention and management. Quantitative findings were supplemented by qualitative interviews, where participants attributed poor NCD service readiness to physical and systemic constraints, including inadequate infrastructure, infrequent training opportunities, high staff turnover, and a lack of guidelines and supplies for NCD management. Additionally, federalization and the COVID-19 pandemic exacerbated existing disparities in NCD service readiness. Despite the challenges, some primary healthcare facilities with better infrastructure and capacity managed to provide NCD services through peer coaching, social health insurance schemes, and local government support. These complementary findings provided a nuanced understanding of the systemic and contextual factors influencing the primary healthcare system’s readiness for NCD prevention and management.

## Discussion

We evaluated the primary healthcare system’s readiness and associated factors for preventing and managing DM, CVDs, and CRDs in Nepal. The average NCD service readiness scores for all three NCDs were below the recommended threshold of 70 [40, 41]; therefore, primary healthcare facilities in general were not ready for NCD management. NCD readiness scores varied by the type of primary healthcare facility, with higher readiness in PHCCs compared to health posts; geographical region, with higher readiness in the hills compared to the mountains; and applicability of separate user fees, with higher readiness in primary healthcare facilities that impose user fees. Qualitative findings aligned with quantitative findings, underscoring inconsistent availability of trained staff, guidelines, diagnostic equipment, and medicines for NCD management in primary healthcare facilities. Additionally, the COVID-19 pandemic and federalization complicated the attainment and allocation of resources for NCD management, but some local municipalities managed to improve access to NCD medicines. Health insurance in PHCCs strengthened NCD service delivery through improved availability of drugs, human resources, and referrals.

In our study, the overall service readiness scores for DM and CVDs were comparatively higher than CRDs, a phenomenon also observed in studies from Bangladesh [29], Kenya [28], and Myanmar [42]. The lower CRD readiness may be due to two reasons. First, DM and CVDs are often prioritized in global and national health strategies due to their high prevalence and the significant burden they impose on healthcare systems. Second, managing CRDs requires advanced equipment and specialized care (e.g., oxygen therapy and pulmonary rehabilitation) that may be difficult to set up and operate at the primary level. This finding, however, is concerning as CRDs are one of the leading causes of mortality in Nepal [43, 44].

Despite the variation in overall service-readiness scores, the availability of trained staff and guidelines was similar for DM, CVDs, and CRDs, possibly because the national PEN manual covers basic precepts for managing all three NCDs [12]. Some PHCCs had staff additionally trained for handling cardiac emergencies, including basic and advanced life support. Yet, one-third of primary healthcare facilities lacked PEN-trained personnel, consistent with studies from other resource-constrained settings, such as Bangladesh [30], India [45], and Vietnam [46]. The unavailability of trained staff was ubiquitous in all primary healthcare facilities of Nepal, primarily due to adjustments following federalism. This emerged prominently in our qualitative interviews, underscoring the need for all levels of government to collaborate closely to track existing PEN-trained staff, train new healthcare staff, and deploy trained healthcare staff prudently. Additionally, PEN-trained staff should take responsibility for PEN orientation and peer coaching for their colleagues. Task shifting should be practiced to avoid NCD service discontinuity in the absence of PEN-trained staff in primary healthcare facilities [47].

Early screening and management of NCDs heavily rely on the availability of testing equipment; however, our study revealed a wide variation in the availability of NCD testing equipment. Basic equipment like weighing scales, stethoscopes, and BP sets was readily available in primary healthcare facilities, similar to studies in Pakistan [48], Tanzania [27], and Cambodia [49]. The uniformity in study findings across countries can be attributed to their utility beyond NCDs, as they are essential in diagnosing and treating all disease conditions. Consistent with a study in Pakistan [48]. Most primary healthcare facilities in our setting lacked functional equipment for diabetes management, possibly due to the high maintenance costs of glucometers and the non-reusable nature of lancets and testing strips. Thus, addressing these gaps in NCD-specific diagnostic equipment requires not only the provision of equipment but also sustainable strategies for maintenance and consistent supply.

Only a few primary healthcare facilities in our study were equipped with peak flow meters for CRD diagnosis, similar to a study in Tanzania [27]. Moreover, primary healthcare facilities lacked peak flow meters, spacers, and metered-dose inhalers (MDIs) to conduct peak flow tests, a recommended diagnostic test for CRDs in the PEN program. The unavailability of diagnostic equipment led health service providers to rely on history taking and physical examination in diagnosing and prescribing medicines for NCD patients. This practice was commonplace in all primary healthcare facilities and could severely compromise the quality of care through inappropriate diagnosis and irrational use of drugs. The prolonged use of unwarranted medication can increase the risk of adverse drug events in NCD patients [50, 51]; therefore, primary healthcare facilities should focus on other objective diagnostic methods to improve the accuracy of diagnosis.

Our study revealed critical gaps in the readiness of primary healthcare facilities to provide essential NCD drugs, with this domain scoring the lowest among all assessed domains. Primary healthcare facilities, particularly health posts, were authorized to dispense limited NCD drugs [52], yet those basic drugs were also unavailable. Nearly half of the primary healthcare facilities lacked amlodipine and metformin, with the lowest availability for atorvastatin. The persistent shortage of NCD drugs in primary healthcare facilities could eventually lead to increased referrals to higher-level centers, overburdening them. Similar to studies in Tanzania [27] and Myanmar [42]. Primary healthcare facilities in our study lacked salbutamol and corticosteroid inhalers. As a result, primary healthcare facilities, mostly health posts, only dispensed salbutamol tablets despite their low effectiveness in treating CRDs. CRD patients were compelled to buy medicines from private drug outlets, which are considerably expensive. Given the chronic nature of NCDs and the requirement for NCD patients to take medication for life-long, the out-of-pocket payments for NCD management can be catastrophic to poor NCD patients inhabiting rural and mountain areas [53, 54]. Consequently, many NCD patients drop out of the treatment plan and turn to unproven conventional treatment practices [55].

Compared to health posts, PHCCs in our study had consistently higher service readiness for all three NCDs. This difference might exist because PHCCs in general are more advanced than health posts in terms of infrastructure and resources, and serve as a referral point for health posts [56]. Another possible reason for improved readiness could be the social insurance coverage in PHCCs. Our qualitative findings also corroborate our reasoning, as several health service providers reported an increased supply of NCD medicines after the liaison with the social health insurance board. Health posts, on the contrary, only dispensed limited NCD drugs listed by the Ministry of Health and Population [57]. Hence, expanding social health insurance schemes to health posts might help initiate the PEN program in health posts, the first point of contact for NCD patients.

Our findings revealed significant regional disparities in the service readiness for CVDs and CRDs, with higher readiness in the hills compared to the mountains and the Lowlands. This variability in service readiness may stem from the differing timelines of PEN program initiation and scale-up across the sampled districts implementing PEN. The PEN program was launched in tandem with the social health insurance program in Ilam and Kailali districts in 2016 [58, 59]. These PEN-piloted districts - Ilam in the hills and Kailali in the lowlands - received immense support from the government and WHO for the program initiation and continue to serve as model districts and hubs for PEN training and scale-up. On the other hand, primary healthcare facilities in the mountains are hard to reach. The high altitude and rugged mountain topography typically impede the timely delivery of medicines and equipment to primary healthcare facilities; the turnover of healthcare staff is also considerably high. Hence, the government should develop and implement preemptive strategies to retain PEN-trained staff in the mountains and expedite the delivery of medical supplies to primary healthcare facilities.

Implementing small user fees for some services (e.g., blood glucose testing and dressing for diabetic foot) significantly improved NCD readiness in primary healthcare facilities in our study. Our finding further expands on Nepal’s national health facility survey results, which reported an average positive association between user fees and NCD readiness [60]. Despite this, given the cross-sectional nature of our data, we cannot conclude whether user fees causally lead to improved NCD service readiness or vice versa. User fees could enhance the autonomy of primary healthcare facilities in purchasing medical supplies and covering additional operational costs. However, caution is necessary when implementing user fees, as they could deter low-income patients from accessing NCD services at primary healthcare facilities. [61].

### Policy implications

We propose three recommendations for all levels of government – federal, provincial, and local.

First, the government should allocate PEN-trained personnel to primary healthcare facilities that experienced a consequential loss of PEN-trained personnel after federalism. The central, provincial, and local training centers could play a pivotal role in identifying primary healthcare facilities lacking a trained workforce. Different media platforms should be leveraged to livestream training sessions for participants who are unable to attend in-person training sessions. Subsequently, training materials and video recordings of training sessions can be used to further develop self-paced online training modules. Training participants should be delegated the responsibility of organizing peer coaching sessions to facilitate knowledge sharing with fellow workers. PEN trainers should also routinely follow up on training participants, evaluate trainees’ performance, and lend supportive supervision. For the long-term availability of PEN-trained staff and sustainable PEN services, the core components of PEN training should be integrated into the training curricula of medical, nursing, and paramedic students.

Second, the government should focus on building multi-sectoral partnerships within and outside the government for supply chain management. The national social health insurance program should be expanded to health posts, and other parallel programs (e.g., the senior citizenship program) should be integrated into the PEN program to escalate program activities.

Third, the government should allocate an adequate budget for printing the PEN manual, CVD risk estimation charts, and other information, education, and communication (IEC) materials related to NCDs. The CVD risk estimation charts and PEN algorithms should be made available as flip charts and posters to aid health service providers during patient examinations. In case of unavailability of hard copies, at least soft copies of CVD risk estimation charts and guidelines should be circulated to health service providers.

### Strengths and Limitations

Our study has several strengths. First, this study is the first to comprehensively evaluate the readiness of primary healthcare facilities across all geographic regions of Nepal. Second, trained research assistants collected data using the adapted WHO-SARA tool and verified the availability of essential items: NCD guidelines, equipment, and medicines. Third, the quantitative estimates were weighted to address the oversampling of PHCCs and the unequal probability of selecting primary healthcare facilities due to the complex survey design. Finally, the qualitative findings support the quantitative results and reveal various contextual factors that contribute to higher or lower NCD service readiness.

Despite its strengths, our study has limitations. First, our study did not account for the design effect inherent in the multistage sampling, which may have led to potentially underestimating the standard errors and confidence intervals. Second, the NCD service readiness was ascertained based on the availability of tracer items and the mere presence of one functional equipment, or an unexpired drug, indicated readiness in primary healthcare facilities. This could overestimate NCD service readiness in understocked primary healthcare facilities. Third, information related to the availability and monitoring of trained staff was collected from the health facility in charge; however, it could not be validated through observation of records in all situations. Therefore, health service providers may have underreported or overreported training and monitoring information due to social desirability, fear of repercussions, or an inability to recall accurate information. Thus, the possibility of information and social desirability bias remains in the study.

## Conclusion

The readiness of the primary health system for services related to DM, CVDs, and CRDs reveals substantial deficits in terms of human resources and the availability of guidelines, equipment, and medications. The shortage of essential drugs was identified as a significant hindrance to the delivery of NCD services. High staff turnover was evident in all primary healthcare facilities, and this issue must be addressed to retain PEN-trained staff in these facilities. The readiness of primary healthcare facilities for managing NCDs varied by topography, with minimal readiness in mountainous regions; type of primary healthcare facility, with poor readiness in health posts; and applicability of user fees, with low readiness in primary healthcare facilities that did not impose user fees. Hence, the government should consider these disparities in the primary healthcare system while allocating healthcare resources and scaling up the PEN program activities. The federal, provincial, and local training centers should collaborate to identify primary healthcare facilities lacking personnel trained in PEN and prioritize them further during training sessions. Moreover, training centers should also provide PEN manuals and CVD risk estimation charts to each participant attending PEN training sessions so they can easily refer to them during clinical practice.

## Supplementary Material

Supplementary Files

This is a list of supplementary files associated with this preprint. Click to download.

- SupplementaryTable1.docx
- SupplementaryTable2.docx
- SupplementaryTable3.docx

## Figures and Tables

**Table 1:** Domains and tracer indicators assessing readiness for the management of DM, CVDs, and CRDs

Domains	Tracer indicators	Assessment
Staff training and guidelines	Guidelines for the diagnosis and management of DM, CVDs, and CRDs	Availability of the PEN manual or any standard treatment hard copy protocols for DM, CVDs, and CRDs in primary healthcare facilities.
	Staff trained in the diagnosis and management of DM, CVDs, and CRDs	Self-reported availability of at least one staff trained in PEN or completion of other relevant courses in diagnosis and management of DM, CVDs, and CRDs.
Equipment and diagnostics	DM: blood pressure (BP) apparatus, adult weighing scale, measuring tape or stadiometer, glucometer, blood glucose test strips, urine dipstick protein, and urine dipstick ketone	Availability of functioning digital or aneroid BP measuring apparatus, adult weighing scale, measuring tape or stadiometer, glucometer, blood glucose test strips, and valid urine strips for the measurement of protein and ketone.
	CVD: BP measuring apparatus, adult weighing scale, stethoscope, measuring tape, and oxygen	Availability of functioning stethoscope, digital or aneroid BP measuring apparatus, adult weighing scale, measuring tape, oxygen, and CVD risk estimation charts.
	CRD: stethoscope, peak flow meter, spacers for inhalers, and oxygen	Availability of functioning stethoscope, peak flow meter, spacers for inhalers, and oxygen
Basic medicines	DM: metformin, sulfonylureas, insulin, dextrose injection	Availability of at least one valid metformin, sulfonylureas, insulin, and dextrose injection.
	CVD: calcium channel blocker, enalapril, furosemide, hydrochlorothiazide, atorvastatin, aspirin, and metformin	Availability of at least one valid calcium channel blocker, enalapril, furosemide, aspirin, hydrochlorothiazide, atorvastatin, and metformin
	CRD: beclomethasone inhaler, salbutamol tablets, prednisolone, hydrocortisone injection, and epinephrine injection	Availability of at least one valid beclomethasone inhaler, salbutamol tablets, prednisolone, hydrocortisone injection, and epinephrine injection

DM: Diabetes Mellitus, CRDs: Chronic Respiratory Diseases, CVDs: Cardiovascular Diseases, PEN: Package of Essential Non-Communicable Diseases

**Table 2:** Background characteristics of primary healthcare facilities in districts where the PEN program was implemented (n=105)

Variable	Unweighted	Weighted
	n (%)	n (%)
**Facility type**		
Health post	71(67.6)	99(94.1)
PHCC	34(32.4)	6(5.9)
**Area**		
Rural	52(49.5)	60(57.1)
Urban	53(50.5)	45(42.9)
**Ecological region**		
Mountain	10(9.5)	7(6.2)
Hills	39(37.1)	43(41.4)
Lowlands	56(53.3)	55(52.4)
**Opening hours, Median (IQR)**	7(7.0, 10.4)	7(7,7)
**Emergency service**		
Available	54(51.4)	37(35.6)
Not available	51(48.6)	68(64.4)
**Health insurance**		
Implemented	30(28.6)	7(6.8)
Not Implemented	75(71.4)	98(93.3)
**User fee for NCD services**		
Yes	54(51.4)	31(29.7)
No	51(48.6)	74(70.3)
**Distance to nearest referral center (in kilometers)**	20(11,30)	16(9,25)
**Monitoring and supervision**		
In the last three months	2(1.9)	1(1.3)
More than three months ago	12(11.4)	15(13.9)
Never performed	91(86.7)	89(84.8)

n= frequency, IQR=Interquartile range (Q_1_, Q_3_), PHCC: Primary Healthcare Centers, IQR consists of the first quartile (Q_1_) and the third quartile (Q_3_)

**Table 3:** Factors associated with primary healthcare facility readiness for the management of NCDs (n=105)

Characteristics	CVD readiness		DM readiness		CRD readiness	
	Unadjusted β (95% CI)	Adjustedβ (95% CI)	Unadjusted β (95% CI)	Adjustedβ (95% CI)	Unadjusted β (95% CI)	Adjustedβ (95% CI)
**Facility type**						
Health post	Ref	Ref	Ref	Ref	Ref	Ref
PHCC	16.52(4.74 to 28.30) *	9.88(0.37 to 19.34) *	21.42(7.23 to 35.61) *	11.90(0.37 to 23.43) *	14.63(2.22 to 27.08) *	13.18(4.96 to 21.40) *
**Location**						
Rural	Ref	Ref	Ref	Ref	Ref	Ref
Urban	−1.62(−15.98 to 12.75)	1.12(−5.44 to 7.68)	0.89(−16.84 to 18.62)	3.76(−7.21 to 14.72)	3.69(−19.87 to 12.49)	−0.37(−8.16 to 7.42)
**Region**						
Mountain	Ref	Ref	Ref	Ref	Ref	Ref
Lowlands	−0.19(−7.73 to 7.34)	−0.21(−6.79 to 6.37)	−8.22(−19.05 to 2.61)	−8.42(−7.21 to 14.72)	−5.31(−14.67 to 4.06)	−5.28(−13.74 to 3.20)
Hill	19.83(13.54 to 26.13) *	17.63(10.18 to 25.08) *	12.84(2.63 to 23.06) *	10.35(−2.18 to 22.89)	17.76(10.50 to 25.02) *	16.43(9.36 to 23.50) *
**Separate fee**						
No	Ref	Ref	Ref	Ref	Ref	Ref
Yes	17.38(8.15 to 26.61) *	11.75(5.05 to 18.44) *	20.09(8.13 to 32.05) *	14.21(5.44 to 22.98) *	13.63(3.02 to 24.23) *	6.79(0.74 to 12.84) *

* Statistically significant at p-values less than 0.05

Ref: Reference category, DM: Diabetes Mellitus, HTN: Hypertension, CVDs: Cardiovascular Diseases. CRDs: Chronic Respiratory Diseases

## Data Availability

The datasets used in this study are not publicly available due to the confidentiality of certain data.

## Abbreviations

CVDs: Cardiovascular diseases
DM: Diabetes Mellitus
CRDs: Chronic Respiratory Diseases
DALYs: Disability Adjusted Life Years
LMICs: Low- and Middle-Income Countries
NCDs: Non-communicable diseases
SARA: Service Availability Readiness Assessment
WHO-PEN: World Health Organization-Package of Essential Non-communicable Diseases
IEC: Information, Education, and Communication
HA: Health Authority
HSP: Health Service Provider
